# Errata

**DOI:** 10.36660/abc.20200016

**Published:** 2020-01

**July 2019 issue, vol. 113 (1), pages 52-59**

In the Original Article " Association of Dietary Patterns with Excess Weight and Body Adiposity in Brazilian Children: The Pase-Brasil Study", in Figure 1, in the chart referring to increased body fat x traditional pattern, consider the sign correct < P75.

**Arq Bras Cardiol. 2019; [online].ahead print, PP.0-0**

In the Review Article “Artificial Intelligence in Cardiology: Concepts, Tools and Challenges - “The Horse is the One Who Runs, You Must Be the Jockey”, in Figure 2, replace the acronym KM with KNN and the acronym DAM with GB.

